# The malate–aspartate shuttle supports thermogenic lipid mobilization in brown adipocytes

**DOI:** 10.1111/febs.70461

**Published:** 2026-02-18

**Authors:** Michaela Veliova, Caroline M. Ferreira, Katrina P. Montales, Francisco Villalobos, Alexandra J. Brownstein, Rebeca Acín‐Pérez, Gabrielly S. Ferreira, Anthony E. Jones, Linsey Stiles, Ajit S. Divakaruni, Marc Liesa, Orian S. Shirihai, Marcus F. Oliveira

**Affiliations:** ^1^ Department of Molecular and Medical Pharmacology, and Division of Endocrinology, David Geffen School of Medicine University of California Los Angeles USA; ^2^ Institute of Medical Biochemistry Leopoldo de Meis Universidade Federal do Rio de Janeiro Brazil; ^3^ Institut de Biologia Molecular de Barcelona IBMB, CSIC Spain; ^4^ Ciberdem Instituto de Salud Carlos III Madrid Spain

**Keywords:** energy, heat, metabolism, obesity, redox, thermogenesis

## Abstract

Brown adipose tissue (BAT) plays a central role in thermogenesis by coupling fatty acid oxidation to heat production. Efficient BAT thermogenic activity requires enhanced glycolytic flux, which in turn depends on continuous regeneration of cytosolic NAD^+^ to sustain glyceraldehyde‐3‐phosphate dehydrogenase activity. This regeneration is mediated by three main pathways: lactate dehydrogenase, the glycerol‐3‐phosphate shuttle (GPSh), and the malate–aspartate shuttle (MASh). We previously showed that inhibition of the mitochondrial pyruvate carrier increases energy expenditure in brown adipocytes via MASh activation. However, the specific contribution of MASh to BAT energy metabolism remains poorly defined. Here, we show that MASh is functional and directly regulates lipid metabolism in BAT. Enzymatic activities of cytosolic and mitochondrial malate dehydrogenases and glutamic–oxaloacetic transaminases in BAT were comparable to those in the liver. Using a reconstituted system of isolated BAT mitochondria and cytosolic MASh enzymes, we demonstrated that extra‐mitochondrial NADH is efficiently reoxidized in a glutamate‐dependent manner via MASh. Genetic silencing of the mitochondrial carriers critical to MASh, namely the oxoglutarate carrier (*Ogc*) and aspartate–glutamate carrier (*Aralar1*), had no apparent effects on respiratory rates. However, silencing either *Ogc* or *Aralar1* led to the accumulation of small lipid droplets and impaired norepinephrine‐induced lipolysis. Taken together, our data indicate a novel role of MASh in regulating BAT lipid homeostasis with potential implications to body energy expenditure and thermogenesis.

## Graphical abstract

Brown fat cells burn lipids within their mitochondria to generate heat. This process involves two energy “shuttles,” one of which is naturally blocked during heat production. We found that the second shuttle (MASh) is not required to generate heat. However, when MASh is disabled, the fatty acids meant for fuel are instead converted back into stored fat. This causes fat accumulation, proving MASh is essential for regulating fat storage.

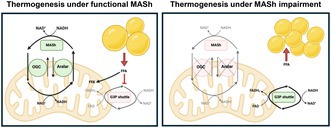

**Abbreviations**
AOA: aminooxyacetate
Aralar1: aspartate‐glutamate carrier 1 (SLC25A12)
Atgl: adipose triglyceride lipase
BAT: Brown Adipose Tissue
Ca^2+^
: Calcium ion
cG3PDH: NAD‐dependent cytosolic glycerol‐3‐phosphate dehydrogenase
CPT1: Carnitine Palmitoyltransferase 1
DHAP: dihydroxyacetone phosphate
Elovl3: very‐ long‐ chain fatty acid elongase 3
FFA: free fatty acid
G3P: glycerol‐3‐phosphate
GAPDH: glyceraldehyde‐3‐phosphate dehydrogenase
GOT: glutamate‐oxaloacetate transaminase
GOT1: cytosolic glutamate‐oxaloacetate transaminase 1
GPSh: glycerol‐3‐phosphate shuttle
HSL: hormone‐sensitive lipase
LD: lipid droplet
LDH: lactate dehydrogenase
MAG: monoacylglycerol
MASh: malate‐aspartate shuttle
MDH: malate dehydrogenase
mG3PDH: FAD‐dependent mitochondrial glycerol‐3‐phosphate dehydrogenase
MPC: mitochondrial pyruvate carrier
MTDR: mitotracker deep red
NAD^+^
: nicotinamide adenine dinucleotide
NADH: nicotinamide adenine dinucleotide
NE: norepinephrine
OCR: oxygen consumption rates
Ogc: oxoglutarate carrier (SLC25A11)
PC: pyruvate carboxylase
Pgc1α: peroxisome proliferator‐activated receptor gamma coactivator 1‐alpha
Prdm16: PR domain containing 16
TAG: triacylglycerol (Triglyceride)
TCA: tricarboxylic acid
Tfam: mitochondrial transcription factor A
TLC: thin‐ layer chromatography
TMRE: tetramethylrhodamine ethyl ester
UCP1: uncoupling protein 1

## Introduction

Brown adipose tissue (BAT) is a specialized thermogenic organ that dissipates energy as heat through a process known as non‐shivering thermogenesis. This function is mediated by uncoupling protein 1 (UCP1), located in the inner mitochondrial membrane, which uncouples oxidative phosphorylation by dissipating the proton gradient. BAT activation in response to cold exposure or adrenergic stimulation triggers lipolysis through enzymes including adipose triglyceride lipase (ATGL) and hormone‐sensitive lipase (HSL) [1, 2]. This results in the release of free fatty acids (FFAs) from lipid droplets, which activate UCP1. In addition, FFAs serve as substrates for β‐oxidation and the tricarboxylic acid (TCA) cycle, thereby increasing nutrient oxidation and mitochondrial oxygen consumption [3]. Given BAT's role in energy dissipation and glucose utilization, understanding its regulatory mechanisms is of growing interest in the context of obesity and metabolic disease [4].

Thermogenically active BAT displays increased glucose uptake and glycolytic flux, both of which require continuous regeneration of cytosolic NAD^+^ for glyceraldehyde‐3‐phosphate dehydrogenase (GAPDH) activity [2, 5]. Disruption of this redox balance impairs glycolysis and thermogenesis, emphasizing the need for effective NAD^+^‐recycling systems [6]. In mammalian cells, cytosolic NAD^+^ is regenerated through lactate dehydrogenase (LDH), the glycerol‐3‐phosphate shuttle (GPSh), or the malate–aspartate shuttle (MASh). In BAT, however, lactate production rises only slightly with adrenergic activation and most lactate is oxidized via the TCA cycle, suggesting that LDH primarily consumes NAD^+^ rather than regenerating it [7, 8, 9, 10]. Consequently, mitochondrial redox shuttles become critical for sustaining cytosolic NAD^+^ supply. These systems bypass the impermeability of the inner mitochondrial membrane to NADH by transferring reducing equivalents into the mitochondrial matrix to support respiration. Among them, the GPSh contributes minimally under thermogenic conditions, as mitochondrial FAD‐dependent glycerol‐3‐phosphate dehydrogenase (mG3PDH) is inhibited by free fatty acids [11].

**Fig. 1:**
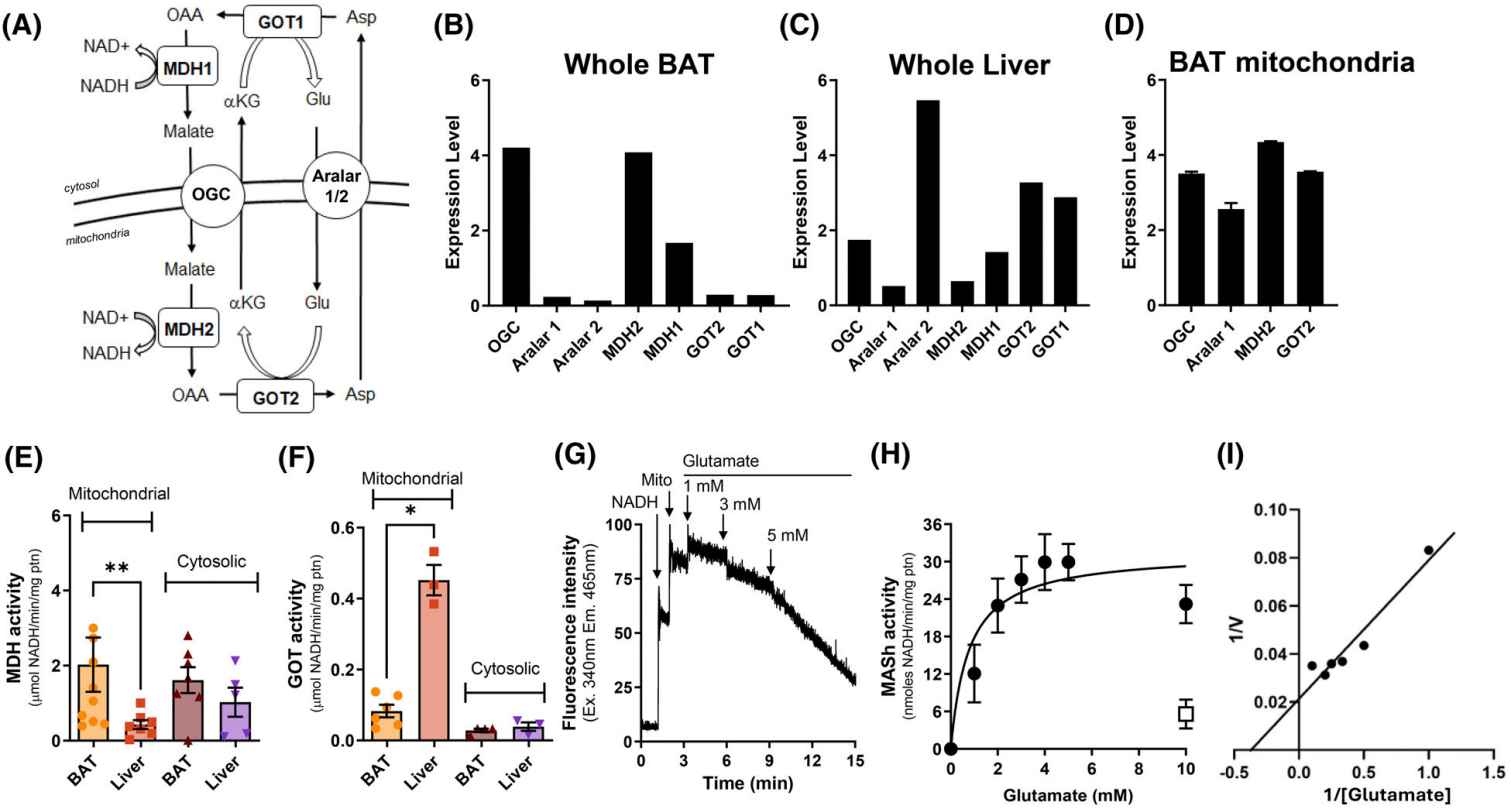
The malate–aspartate shuttle is functional in murine brown adipose tissue. (A) Schematic representation of the MASh. MDH1, malate dehydrogenase 1 (cytosolic), MDH2, malate dehydrogenase 2 (mitochondrial); GOT1, glutamic oxaloacetic transaminase (cytosolic); GOT2, glutamic oxaloacetic transaminase (mitochondrial); OGC (SLC25A11), oxoglutarate carrier; Aralar or Citrin (aspartate glutamate carrier or SLC25A12 or SLC25A13, respectively) which exchanges aspartate and glutamate through inner mitochondrial membrane; NAD+, nicotinamide adenine dinucleotide (oxidized form); NADH, nicotinamide adenine dinucleotide (reduced form). (B–D) *In silico* data mining analysis of the protein components of MASh whole BAT (B), and whole liver (C) determined in quantitative proteomic analyses conducted in [12]. Both cytosolic and mitochondrial components are included. MDH1, malate dehydrogenase 1 (cytosolic); MDH2, malate dehydrogenase 2 (mitochondrial), GOT1, glutamic oxaloacetic transaminase (cytosolic); GOT2, glutamic oxaloacetic transaminase (mitochondrial); OGC, oxoglutarate carrier; Aralar 1, aspartate glutamate carrier 1; Citrin, aspartate glutamate carrier 2. (D) *In silico* data mining analysis of the protein components of MASh in BAT mitochondria determined in [13]. Only mitochondrial components are shown (OGC, Aralar 1, MDH2, GOT2); Citrin was not detected in this dataset. MDH2, malate dehydrogenase 2; GOT2, glutamic oxaloacetic transaminase; OGC, oxoglutarate carrier; Aralar 1, aspartate glutamate carrier 1. (E) Enzymatic activity measurements of MDH1 and MDH2 in cytosolic and mitochondrial fractions from BAT and liver. Data are expressed as mean ± SEM from 5 individual experiments with 1–2 replicates each. **P < 0.01 by Mann–Whitney test. (F) Enzymatic activity measurements of GOT1 and GOT2 in cytosolic and mitochondrial fractions from BAT and liver. Data are expressed as mean ± S.E.M. from 3 individual experiments with 1–2 replicates each. *P < 0.05 by Mann–Whitney test. (G) Representative experiment depicting the changes in NADH fluorescence intensity during activation of MASh in isolated BAT mitochondria. Data represent a single experiment that was reproduced in seven independent experiments. (H) MASh activity in BAT mitochondria in the presence of fluorocitrate (black circles) and fluorocitrate + aminooxyacetic acid (AOA) (white square). Data are expressed as mean ± S.D. of seven different experiments. (I) Lineweaver‐Burke plot of data presented in (H).

The MASh is a conserved cyclic redox pathway that circumvents the mitochondrial inner membrane's impermeability to NADH by facilitating the transfer of reducing equivalents from the cytosol to the mitochondrial matrix through the coordinated action of two malate dehydrogenases (MDH), two glutamate‐oxaloacetate transaminases (GOT), and two mitochondrial carriers (Fig. 1A) [14]. MASh operates by first using cytosolic NADH to reduce oxaloacetate to malate, a reaction catalyzed by MDH1. The resulting malate enters the mitochondrial matrix via the malate‐alpha‐ketoglutarate carrier (OGC/SLC25A11). Inside the matrix, MDH2 oxidizes malate back to oxaloacetate, transferring the electrons to mitochondrial NAD^+^ to form NADH. To maintain the cycle, oxaloacetate and glutamate are converted to aspartate and α‐ketoglutarate by GOT2. These products are then transported back into the cytosol via the aspartate–glutamate carrier (Aralar1/SLC25A12) and OGC, where cytosolic glutamate‐oxaloacetate transaminase 1 (GOT1) activity regenerates oxaloacetate from aspartate (Fig. 1A). By transferring electrons from glycolytic NADH to the electron transport system, the MASh enhances OXPHOS and contributes to maintaining a reduced NAD pool within the mitochondria [14, 15, 16, 17]. Although MASh is a well‐characterized metabolic pathway in many mammalian tissues [14, 18], its specific function in BAT is not fully understood. This is particularly significant because BAT utilizes both lipids and glucose for thermogenesis, a process that demands strict control of the cellular redox state. Early studies demonstrated that MASh contributes to increased metabolic activity in BAT after birth, without being influenced by dietary protein intake [18]. Interestingly, cold exposure elevates the expression of key MASh components, including cytosolic and mitochondrial MDH and GOT1 [19]. More recent evidence demonstrates that induction of GOT1 by cold is a key mechanism for MASh activation in BAT, as this activation enhances fatty acid oxidation. Despite reduced glucose oxidation, increased glycolytic flux and glucose uptake support lipid utilization by providing oxaloacetate via pyruvate carboxylase (PC) during cold conditions [9, 20].

Our previous work demonstrated that inhibiting the mitochondrial pyruvate carrier (MPC) in brown adipocytes increases mitochondrial respiration by activating the MASh [21]. This aligns with recent findings where MASh activation, via GOT1 overexpression, shifted cellular energy use from glucose to fatty acid oxidation [20]. Both observations suggest that MASh can compensate for metabolic restrictions by increasing the transfer of NADH into the mitochondria.

Based on this, we hypothesized that MASh plays a role in BAT metabolism beyond simply maintaining redox balance, potentially regulating lipid handling. In this study, we demonstrate that MASh is active in brown adipocytes and is crucial for lipid homeostasis by managing the balance between triglyceride storage and breakdown. While disrupting MASh at different points did not alter overall respiration in brown adipocytes, it triggered compensatory mitochondrial biogenesis and changed the dynamics of lipid droplets. These results establish MASh as a significant regulator of BAT metabolism and suggest that boosting its activity could be a promising strategy for improving metabolic health.

## Results

### The malate–aspartate shuttle is functional in murine brown adipose tissue

We previously demonstrated that pharmacological or genetic inhibition of the mitochondrial pyruvate carrier (MPC) in brown adipocytes enhances energy expenditure through the activation of lipid cycling and the MASh. Specifically, increased mitochondrial respiration under MPC inhibition was attenuated when expression of the MASh transporters, oxoglutarate carrier (*Ogc*) and *Aralar 1*, were downregulated, indicating that MASh is metabolically active in brown adipocytes [21]. Although earlier studies in rats also identified the activity of MASh enzymes in BAT [19], MASh's broader significance and specific functions in BAT metabolism remain largely unexplored.

To characterize the presence and expression of MASh components in BAT, we first conducted an *in silico* analysis using a tissue‐specific quantitative proteomic dataset from C57BL/6J mice [12, 13]. We detected all six canonical components of the MASh in both brown adipocytes as well as in isolated mitochondria (Fig. 1B,D). Importantly, their expression levels were comparable to those found in the liver (Fig. 1C), a tissue where MASh function is well‐established [22].

To determine if MASh is functional in BAT, we measured the enzymatic activities of MDH and GOT in both cytosolic and mitochondrial fractions. Using liver tissue as a positive control, we found that mitochondrial MDH (MDH2) activity was approximately three‐fold higher in BAT than in liver, whereas mitochondrial GOT (GOT2) activity was significantly lower (Fig. 1E,F). The cytosolic activities of both enzymes were comparable between the two tissues. These data support the existence of a functional MASh system in brown adipocytes.

To directly assess the functionality of MASh, we utilized a modified version of previously established methods [18, 23, 24]. This technique involves isolated BAT mitochondria reconstituted with the cytosolic components of the shuttle: MDH, GOT, NADH, aspartate, and glutamate. This experimental setup allows for the measurement of extra‐mitochondrial NADH oxidation over time. The addition of glutamate initiates the cycling of metabolites, facilitating the transfer of reducing equivalents from the cytosol to the mitochondria via the MASh [24]. Fig. 1G shows that sequential additions of glutamate drive extra‐mitochondrial NADH oxidation in a dose‐dependent manner. Glutamate titration revealed a classical Michaelis–Menten saturable curve, with an ^app^ *V*_max_ of 31.5 nmol NADH/min/mg protein and an ^app^ *K*_m_ of 0.77 mm glutamate (Fig. 1H,I). These kinetic parameters were in the same range as those reported for mice brains in the absence of Ca^2+^ (^app^ *V*_max_ = 38 nmol NADH/min/mg protein and ^app^ *K*_m_ of 2.7 mm glutamate) [24, 25]. Glutamate‐induced NADH oxidation was strongly inhibited by the classical transaminase inhibitor aminooxyacetate (AOA), reinforcing the functionality of MASh in BAT (Fig. 1H). Collectively, these data directly demonstrate that MASh is active in BAT and unequivocally demonstrate its ability to transfer cytosolic reduced equivalents of NADH to brown adipocyte mitochondria.

### Brown adipocyte differentiation is preserved following *Aralar 1* and *Ogc* knockdown

**Fig. 2:**
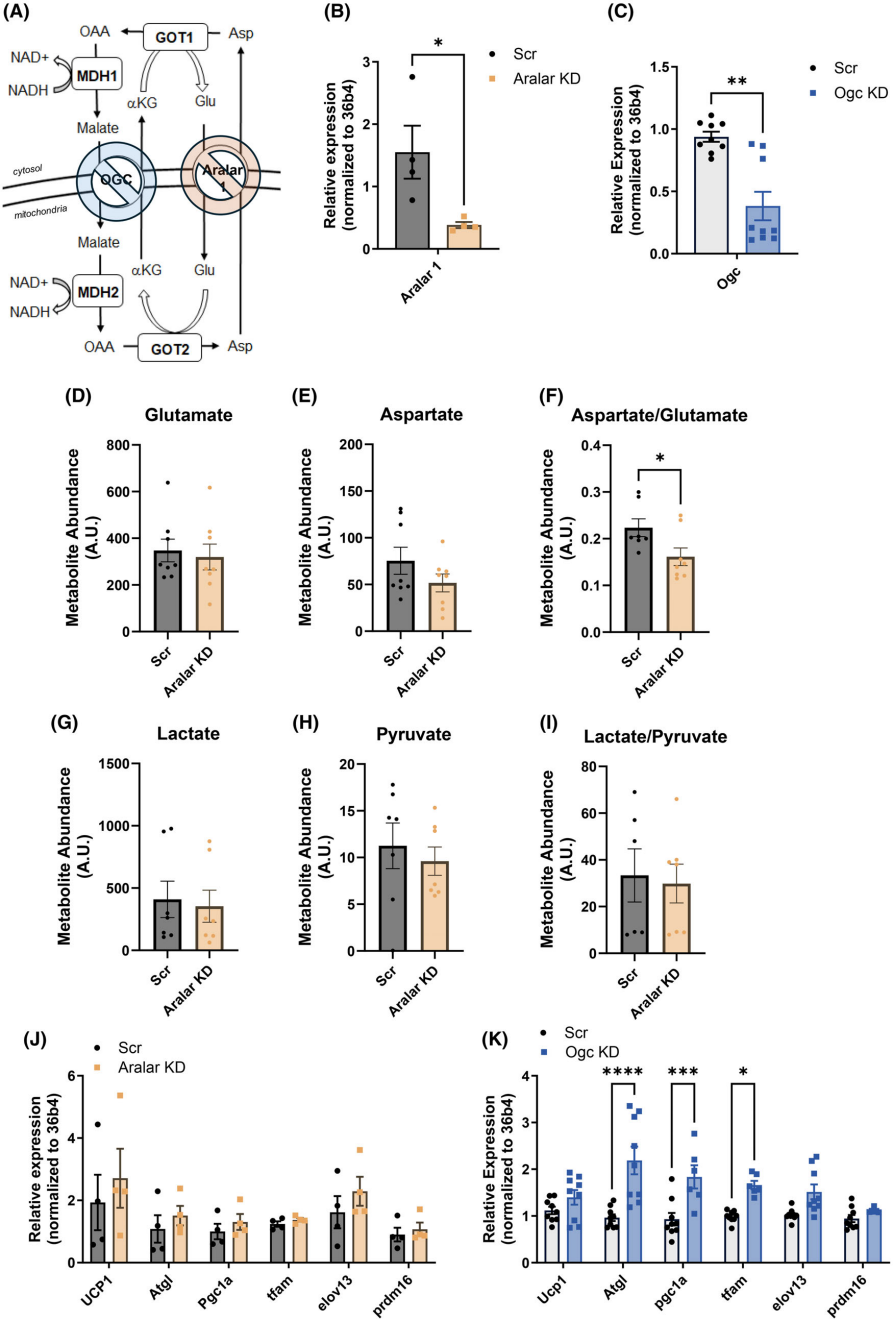
MASh disruption does not affect brown adipocyte cytosolic redox balance and differentiation. (A) Schematic representation of the malate aspartate shuttle (MASh) and the two key mitochondrial transporters genetically silenced in this study. Adenovirus‐mediated shRNA was used to silence *Ogc* (in blue), and siRNA transfection was used to silence *Aralar 1* (in orange). (B) mRNA levels of *Aralar 1* in brown adipocytes transfected with scramble RNA (Scr) or *Aralar 1* siRNA (*Aralar* KD). mRNA levels were normalized to 36b4. Data are expressed as mean ± S.E.M. from *n* = 4 individual experiments. *P < 0.05 by Mann–Whitney test. (C) mRNA levels of *Ogc* in brown adipocytes transduced with adenovirus containing scramble RNA (Scr) or shRNA for *Ogc* (*Ogc* KD). mRNA levels were normalized to 36b4. Data are expressed as mean ± S.E.M. from *n* = 3 individual experiments with 2–3 technical replicates each. **P < 0.01 by Mann–Whitney test. (D–I) Effect of *Aralar 1* KD on polar metabolite abundance quantified using GC–MS. Data are expressed as mean ± S.E.M. of metabolite abundance as arbitrary units (A.U.). *P* values determined by Mann–Whitney test. (D) Glutamate abundance in brown adipocytes transfected with scramble RNA (Scr) or *Aralar 1* siRNA (*Aralar* KD) from *n* = 8 technical replicates performed in 3 individual experiments. (E) Aspartate abundance in brown adipocytes transfected with scramble RNA (Scr) or *Aralar 1* siRNA (*Aralar* KD) from *n* = 8 technical replicates performed in 3 individual experiments. (F) Effect of *Aralar* KD on aspartate/glutamate ratio. Aspartate/glutamate ratio was quantified as a measure of glutamine catabolism from *n* = 7–8 technical replicates performed in 3 individual experiments. *P < 0.05 by Mann–Whitney test. (G) Lactate abundance in brown adipocytes transfected with scramble RNA (Scr) or *Aralar 1* siRNA (*Aralar* KD) as by GC–MS from *n* = 6–7 technical replicates performed in 3 individual experiments. (H) Pyruvate abundance in brown adipocytes transfected with scramble RNA (Scr) or *Aralar 1* siRNA (*Aralar* KD) as by GC–MS from *n* = 6–7 technical replicates performed in 3 individual experiments. (I) Lactate/pyruvate ratio on *Aralar* KD brown adipocytes as a measure of cytosolic NAD redox state. *n* = 6–7 technical replicates performed in 3 individual experiments. (J) mRNA levels of *Ucp1*, *Atgl*, *Pgc1α*, *Tfam*, *Elovl3* and *Prdm16* in brown adipocytes transfected with scramble RNA (Scr) or *Aralar 1* siRNA (*Aralar* KD). mRNA levels were normalized to *36b4*. Data are expressed as mean ± S.E.M. from *n* = 4 individual experiments. (K) mRNA levels of *Ucp1*, *Atgl*, *Pgc1α*, *Tfam*, *Elovl3* and *Prdm16* in brown adipocytes transduced with adenovirus containing scramble RNA (Scr) or shRNA for *Ogc* (*Ogc* KD). mRNA levels were normalized to 36b4. Data are expressed as mean ± S.E.M. from *n* = 3 individual experiments with 2–3 technical replicates each. ****P < 0.0001, ***P < 0.001, **P < 0.01, *P < 0.05 by two‐way ANOVA.

We next investigated the role of the MASh in brown adipocyte energy expenditure during thermogenic stimulation. To accomplish this, we silenced the two mitochondrial carriers essential for the MASh, *Ogc* and *Aralar 1*, in primary brown adipocytes using adenovirus‐mediated shRNA and siRNA, respectively (Fig. 2A). The knockdown efficiency for *Aralar 1* (*Aralar* KD) was approximately 80%, confirmed by qPCR (Fig. 2B). Similarly, adenoviral‐mediated knockdown of *Ogc* (*Ogc* KD) achieved an approximate 80% reduction compared to cells transduced with scramble control (Fig. 2C). To functionally confirm the genetic disruption of MASh, we performed metabolomic analyses to assess the total abundance of several metabolites (Fig. 2D–I). We observed a significant reduction in the aspartate/glutamate ratio in *Aralar 1* KD cells (Fig. 2F), strongly indicating impaired MASh function [26, 27]. Despite this MASh impairment, the cytosolic NAD redox balance remained unaffected, as evidenced by the preserved lactate/pyruvate ratio in *Aralar 1* KD cells (Fig. 2G–I). This suggests the engagement of a compensatory mechanism to maintain cytosolic NAD redox balance. LDH is a natural candidate for such a mechanism, as observed in other models of MASh impairment [28, 29]. However, this does not appear to be the case, as MASh disruption did not significantly alter the abundance of lactate in *Aralar* KD cells, suggesting that mechanisms other than LDH might be responsible for controlling NAD redox balance in this context.

Expression levels of six key differentiation markers were examined to determine whether *Aralar* 1 or *Ogc* knockdown affects brown adipocyte differentiation. Most markers, including *Ucp1*, very long chain fatty acid elongase 3 (*Elovl3*), and PR domain containing 16 (*Prdm16*), remained unchanged (Fig. 2J,K). However, *Ogc* knockdown led to significant increases in adipose triglyceride lipase (*Atgl*), peroxisome proliferator‐activated receptor gamma coactivator 1‐alpha (*Pgc1α*), and mitochondrial transcription factor A (Tfam) mRNA levels, indicating partial reprogramming of the differentiation pathway in these cells (Fig. 2K).

### Genetic ablation of malate–aspartate‐shuttle increases mitochondrial content while preserving brown adipocyte energy expenditure

**Fig. 3:**
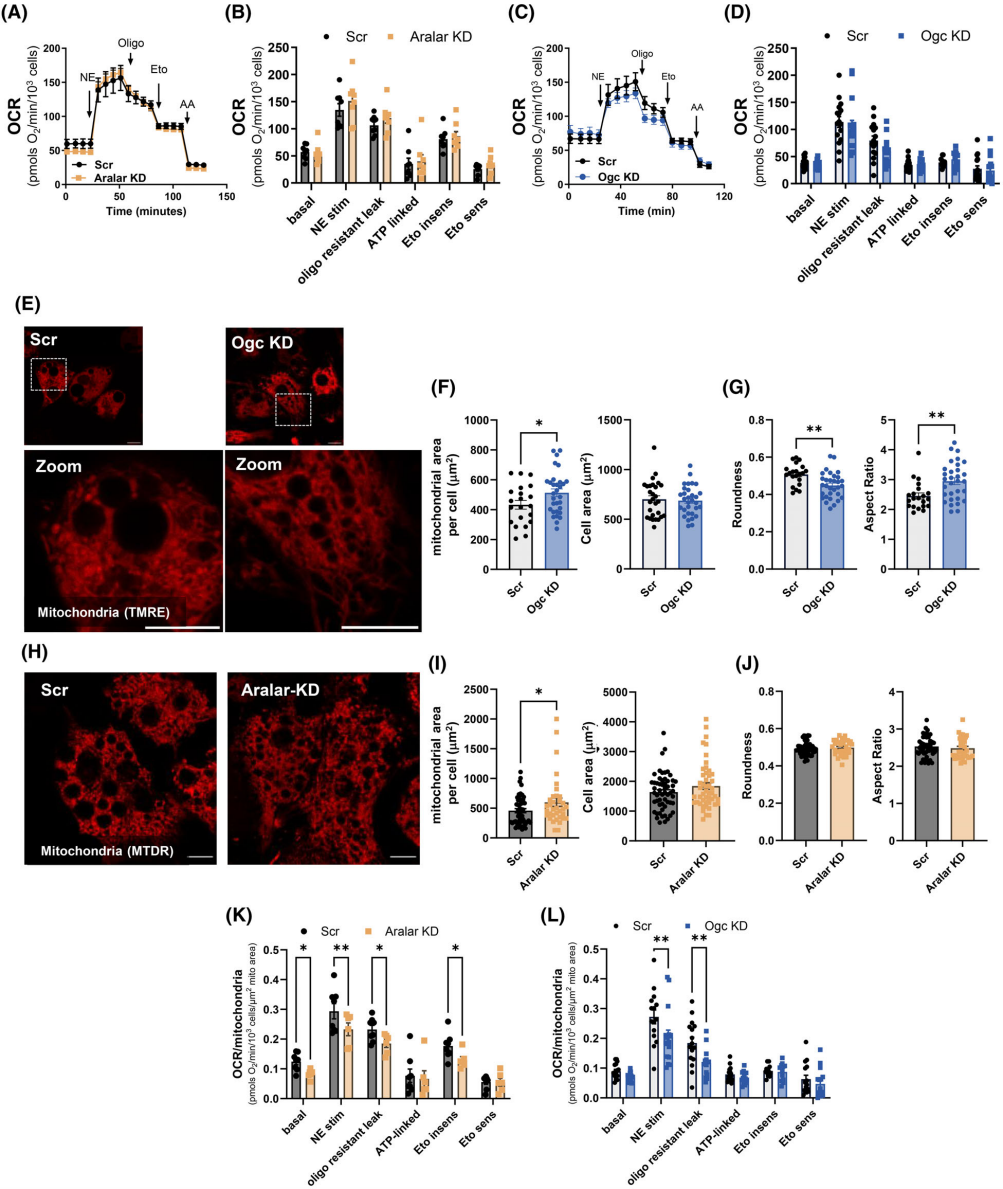
MASh disruption increases mitochondrial content but does not affect energy expenditure in activated brown adipocytes. (A and B) Effect of *Aralar 1* silencing on brown adipocyte energy expenditure. Absolute oxygen consumption rates (OCR) were measured in respirometry media supplemented with 5 mm glucose and 3 mm glutamine. Norepinephrine (NE; 1 μm), oligomycin A (Oligo; 4 μm), etomoxir (Eto; 40 μm) and antimycin A (AA; 4 μm) were injected where indicated. (A) Representative OCR traces averaging 4 technical replicates. Data are expressed as mean ± S.E.M. (B) Quantification of non‐stimulated, NE‐stimulated, oligomycin‐resistant leak, ATP‐linked respiration, etomoxir sensitive and insensitive OCR from *n* = 8 individual experiments. Data are expressed as mean ± S.E.M. (C and D) Effect of *Ogc* KD on brown adipocyte energy expenditure. Absolute OCR was measured in respirometry media supplemented with 5 mm glucose and 3 mm glutamine. NE (1 μm), Oligo (4 μm), Eto (40 μm) and AA (4 μm) were injected where indicated. (C) Representative OCR traces averaging 4 technical replicates. Data are expressed as mean ± S.E.M. (D) Quantification of non‐stimulated, NE‐stimulated, oligomycin‐resistant leak, ATP‐linked respiration, etomoxir sensitive and insensitive OCR from *n* = 8 individual experiments. Data are expressed as mean ± S.E.M. (E) Representative live‐cell confocal images of primary brown adipocytes transduced with adenovirus containing either Scramble RNA (Scr) or shRNA for *Ogc* (*Ogc* KD). Mitochondria were stained with 15 nm TMRE. The scale bar represents 10 μm. (F) Quantification of mitochondrial area per cell, and average cell area from images shown in (E). Data represents *n* = 29–31 cells from 3 individual experiments. Data are expressed as mean ± S.E.M. *P < 0.05 by unpaired *t*‐test. (G) Quantification of the effects of *Ogc* KD on mitochondrial morphology parameters (roundness and aspect ratio). Data represents *n* = 24–31 cells from 3 individual experiments. Data are expressed as mean ± S.E.M. **P < 0.01 by unpaired *t*‐test. (H) Representative live‐cell super‐resolution confocal images of primary brown adipocytes transfected with either Scramble RNA (Scr) or siRNA for *Aralar 1* (*Aralar* KD). Mitochondria were stained with 500 nm mitotracker deep red (MTDR) prior to imaging. The scale bar represents 10 μm. (I) Quantification of mitochondrial area per cell, and average cell area from images shown in (H). Data represents *n* = 45–55 cells from 3 individual experiments. Data are expressed as mean ± S.E.M. *P < 0.05 by unpaired *t*‐test. (J) Quantification of the effects of *Aralar 1* silencing on mitochondrial morphology parameters (roundness and aspect ratio). Data represents *n* = 54–55 cells from 3 individual experiments. Data are expressed as mean ± S.E.M. (K and L) Quantification of the respiratory states determined in *Aralar 1* (A and B) and *Ogc* (C and D) silenced brown adipocytes normalized by their respective mitochondrial content (I and F). Data are expressed as mean ± S.E.M. **P < 0.01, *P < 0.05 by two‐way ANOVA.

To determine whether *Aralar 1* or *Ogc* KD affects energy expenditure, we assessed respiration of intact brown adipocytes using Seahorse XF24 extracellular flux analysis. Oxygen consumption rates (OCR) measured by Seahorse provide a reliable proxy for cellular energy expenditure, as mitochondrial respiration drives most energy use in BAT. This method enables real‐time assessment of mitochondrial function in intact cells with minimal stress. Measurements were performed in media containing 5 mm glucose and 3 mm glutamine and included basal respiration followed by norepinephrine (NE) stimulation to assess activated metabolic states. Glutamine concentration was chosen according to our previously established protocols [21], given its essential role as a precursor for glutamate, a key metabolite in the MASh pathway. Moreover, carnitine palmitoyltransferase 1 (CPT1) inhibitor etomoxir was injected to assess the proportion of OCR that is dependent on fatty acid oxidation. Surprisingly, neither *Aralar 1* nor *Ogc* KD altered the mitochondrial metabolic parameters assessed (Fig. 3A–D). These results underscore that the brown adipocyte differentiation program was not altered by the genetic interventions, since the level of NE‐induced activation of oxygen consumption in both *Aralar 1* and *Ogc* KD was unchanged as previously indicated (Fig. 2J,K). These findings indicate that MASh is dispensable for maintaining energy expenditure in activated brown adipocytes, possibly due to compensatory mechanisms.

Given that expression of *Tfam* and *Pgc1α*, markers of mitochondrial biogenesis, increased in *Ogc* KD brown adipocytes (Fig. 2K), we next examined whether mitochondrial content and morphology were affected. To accomplish this, we stained non‐stimulated brown adipocytes with either TMRE (for *Ogc* KD cells) or mitotracker deep red (MTDR, for *Aralar 1* KD cells) and observed the fluorescence pattern by confocal microscopy (Fig. 3E,H) followed by digital image analyses. We observed that both *Ogc* and *Aralar 1* KD cells had increased mitochondrial area per cell compared with each of their scramble controls, without changing the total cell size (Fig. 3F,I). Interestingly, we also found that mitochondria of *Ogc* KD cells had increased aspect ratio and reduced roundness, indicative of mitochondrial elongation (Fig. 3G). However, we did not observe differences in mitochondrial morphology in *Aralar 1* KD cells compared to their controls (Fig. 3J).

Together our results indicate that while the MASh is active in brown adipocytes, its disruption does not impair mitochondrial oxygen consumption. Instead, MASh‐deficient cells seem to initiate a compensatory increase in mitochondrial biogenesis, likely to maintain thermogenic capacity by overcoming the reduced transfer of NADH equivalents. Indeed, knockdowns of either *Ogc* or *Aralar 1* KD reduced NE‐stimulated and oligomycin‐resistant oxygen consumption per organelle when normalized to mitochondrial content (Fig. 3K,L). This further supports the compensatory mitochondrial biogenesis triggered by disruption of the MASh. The altered mitochondrial morphology in *Ogc* KD cells (Fig. 3G) further suggests that these newly produced mitochondria are metabolically rewired, potentially due to changes in fuel availability [30].

### MASh disruption increases lipid droplet content and drives triglyceride storage in brown adipocytes

**Fig. 4:**
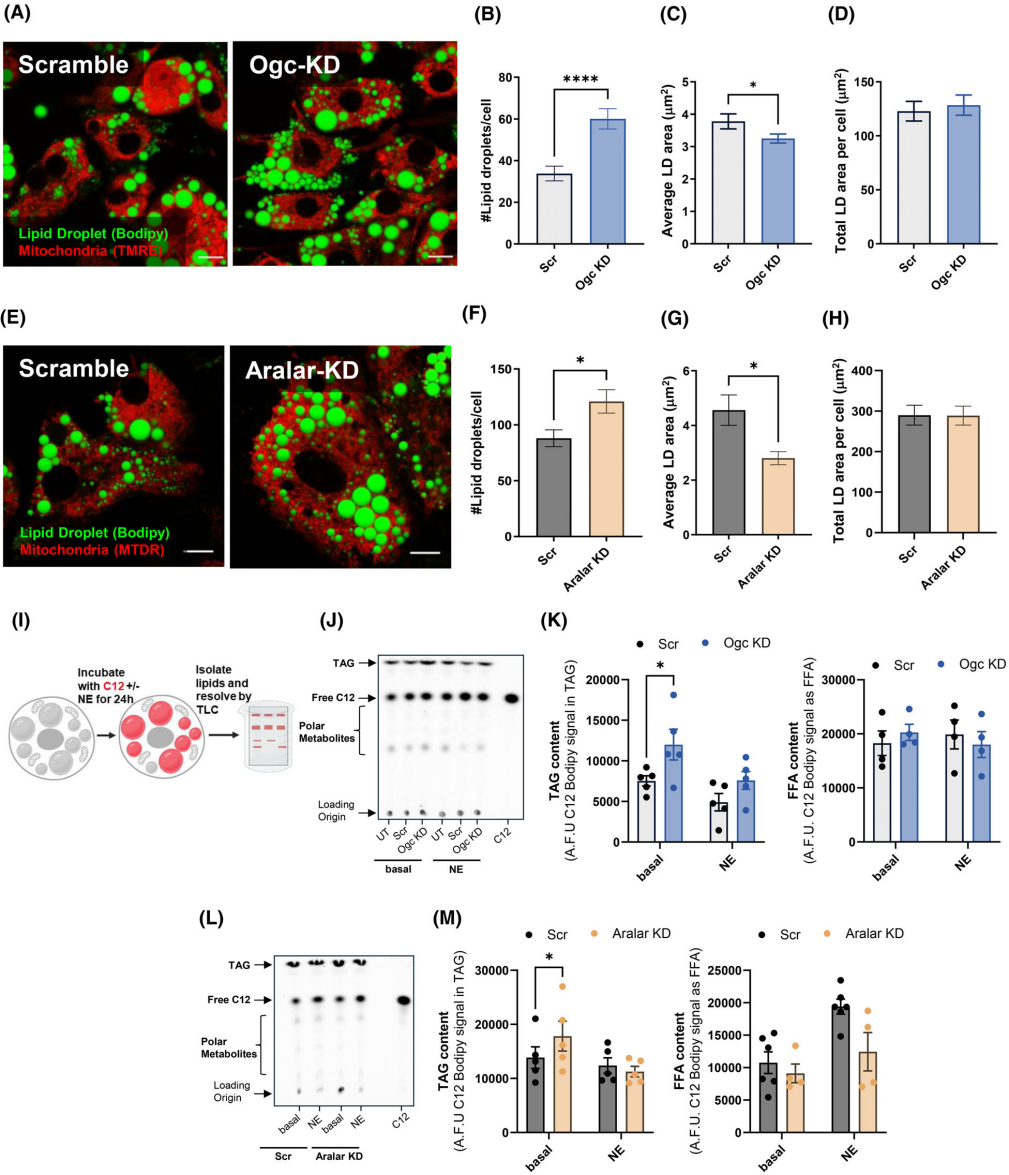
MASh regulates brown adipocytes triglyceride storage. (A) Representative live‐cell confocal images of primary brown adipocytes transduced with adenovirus containing either Scramble RNA (Scr) or shRNA for *Ogc* KD. Lipid droplets (LD) were stained with BODIPY 493 and mitochondria were stained with TMRE prior to imaging. The scale bar represents 10 μm. (B–D) Quantification of number of LD per cell, average LD area per cell, and total LD area per cell from multiple cells under the same conditions as images shown in (A). Data are expressed as mean ± S.E.M. and represent *n* = 22–34 cells from 3 individual experiments. ****P < 0.0001, *P < 0.05 unpaired *t*‐test. (E) Representative live‐cell super‐resolution confocal images of primary brown adipocytes transfected with either Scramble RNA (Scr) or siRNA for *Aralar 1* (*Aralar* KD). Lipid droplets (LD) were stained with BODIPY 493 and mitochondria were stained with mitotracker deep red (MTDR) prior to imaging. The scale bar represents 10 μm. (F–H) Quantification of number of LD per cell, average LD area per cell, and total LD area per cell from multiple cells under the same conditions as images shown in (E). Data are expressed as mean ± S.E.M. and represent *n* = 45–55 cells from 3 individual experiments. **P < 0.01, *P < 0.05 unpaired *t*‐test. (I) Schematic representation of the experimental design for Figures J–M. Cells were incubated with BODIPY C12 (C12) for 24 h in the presence and absence of 1 μm NE. Lipids were extracted from the cells and resolved by thin‐layer chromatography (TLC). (J) Representative TLC plate of lipids extracted from primary brown adipocytes that were untransduced (UT) or transduced with adenovirus containing either Scramble RNA (Scr) or shRNA for *Ogc* (*Ogc* KD), in the presence and absence of NE. C12 alone was loaded as control for free fatty acid. (K) Quantification of TAG from TLC samples shown in (J), and expressed as mean ± S.E.M. of arbitrary fluorescence units (A.F.U.), *n* = 5 individual experiments. *P < 0.05 by two‐way ANOVA. (L) Representative TLC plate of lipids extracted from primary brown adipocytes transfected with either Scramble RNA (Scr) or siRNA for *Aralar 1* (*Aralar* KD) in the presence and absence of NE. C12 alone was loaded as control for free fatty acid. (M) Quantification of TAG from TLC samples shown in (H), and expressed as mean ± S.E.M. of arbitrary fluorescence units (A.F.U.), *n* = 4–6 individual experiments. *P < 0.05 by two‐way ANOVA. Illustrations were created in BioRender.

Given that *Ogc* KD upregulated *Atgl* and *Pgc1α* expression, we next investigated whether MASh impacts lipid metabolism. BODIPY 493/503 staining revealed that *Ogc* KD cells contained significantly more lipid droplets (LDs) than scramble transduced cells (Fig. 4A,B). Interestingly, LDs in *Ogc* KD cells were significantly smaller in size compared to control (Fig. 4C). The total LD area, a surrogate of cellular lipid content, was not changed upon *Ogc* KD (Fig. 4D). Importantly, when *Aralar 1* was silenced in brown adipocytes, we observed similar changes in LD number and size (Fig. 4E–H). This strongly indicates that MASh regulates LD dynamics in brown adipocytes.

Lipid droplet fragmentation can result from either increased triglyceride (TAG) synthesis or enhanced TAG breakdown. To test for increased esterification, we traced incorporation of a fluorescent fatty acid analog (BODIPY‐C12) into lipids (Fig. 4I). After a 24 h pulse and lipid extraction followed by thin‐layer chromatography (TLC), both *Aralar 1* and *Ogc* KD cells showed elevated basal TAG content (Fig. 4J–M). Interestingly, under NE stimulation, TAG levels were similar across control, *Aralar 1*, and *Ogc* KD cells. These results suggest that under non‐stimulated conditions, the MASh limits fatty acid esterification.

### MASh activity is essential for thermogenically activated lipolysis

**Fig. 5:**
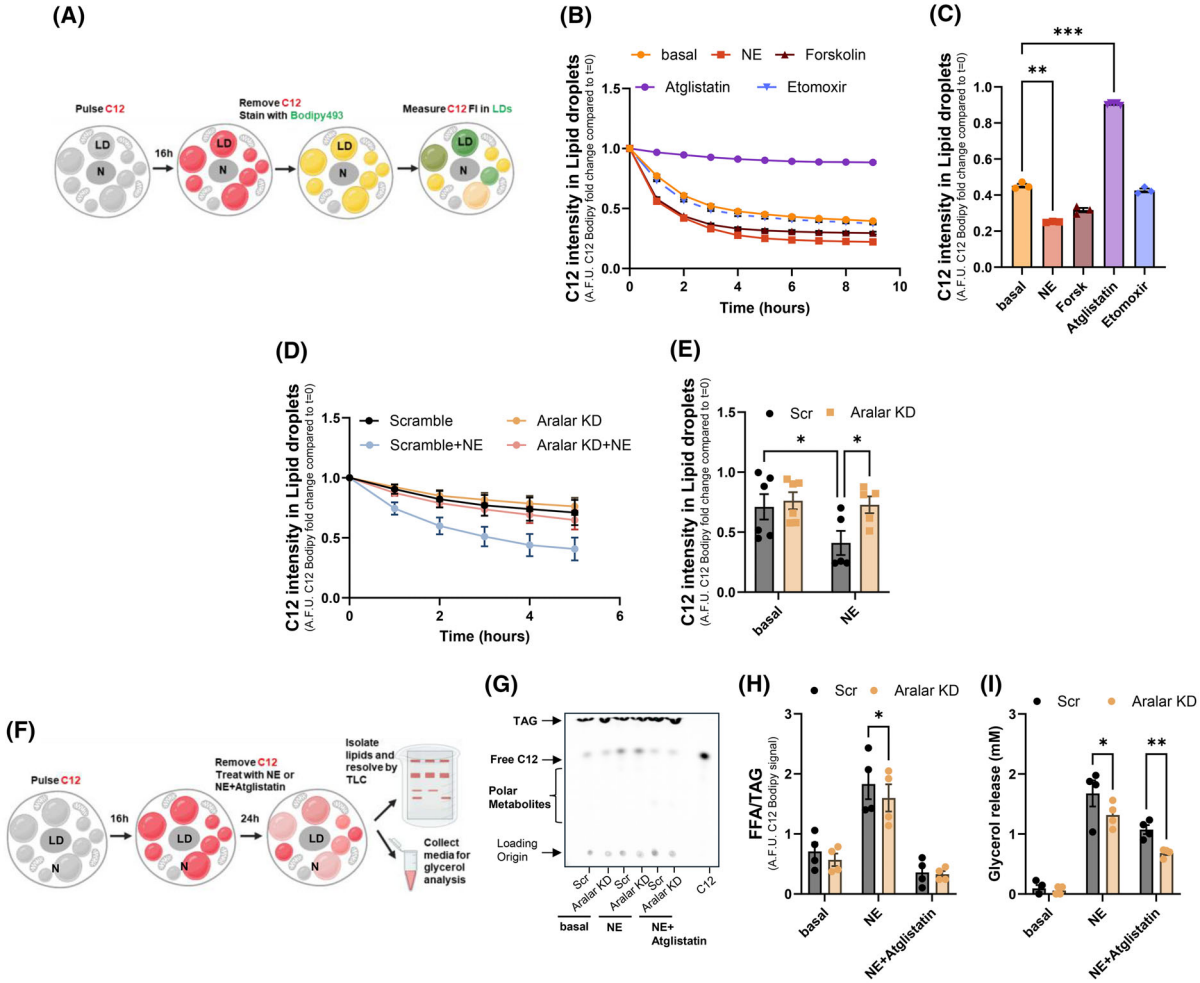
Thermogenically activated lipolysis depends on MASh activity. (A) Schematic representation of the experimental design for panels B‐E. Primary brown adipocytes were incubated with BODIPY C12 (C12) for 16 h to allow enough time for C12 esterification into TAGs. C12 was washed out and replaced by fresh media containing BODIPY 493 which stains all neutral lipids. Cells were imaged once per hour for a time‐course of 5–9 h on Operetta High‐Content Imaging System. (B) Quantification of BODIPY C12 fluorescence intensity (FI) in LDs, as determined by BODIPY 493 staining. Primary brown adipocytes were treated with either 1 μm NE, 10 μm Forskolin, 40 μm Atglistatin, or 10 μm Etomoxir. NE and Forskolin are known inducers of lipolysis in brown adipocytes, Atglistatin blocks lipolysis, and Etomoxir blocks fatty acid oxidation. Note that C12 signal decays faster in conditions where lipolysis is activated. Data are expressed as mean ± S.E.M. of arbitrary fluorescence units (A.F.U.) normalized to *t* = 0. Results reflect a representative experiment with 4 technical replicates. (C) Quantification of C12 FI in LDs after 5 h of treatment. Data are expressed as mean ± S.E.M. of arbitrary fluorescence units (A.F.U.) normalized to *t* = 0 and represent 3–4 independent biological replicates. ***P < 0.001, **P < 0.01 by One‐way ANOVA. (D) Quantification of BODIPY C12 fluorescence intensity (FI) in LDs, as determined by BODIPY 493 staining. Primary brown adipocytes were transfected with either Scramble RNA (Scr) or siRNA for *Aralar 1* (*Aralar* KD) and imaged in the presence and absence of 1 μm NE. Data are expressed as mean ± S.E.M. of arbitrary fluorescence units (A.F.U.) normalized to *t* = 0. Results reflect a representative experiment averaging 3 technical replicates. (E) Data shows C12 FI in lipid droplets after 5 h of treatment and was normalized to *t* = 0. Data are expressed as mean ± S.E.M. and represent *n* = 5–6 technical replicates from 2 individual experiments. *P < 0.05 by two‐way ANOVA. (F) Schematic representation of the experimental design for panels G‐I. Primary brown adipocytes were transfected with either Scramble RNA (Scr) or siRNA for *Aralar 1* (*Aralar* KD). Cells were incubated with BODIPY C12 (C12) for 16 h to allow enough time for C12 esterification into TAGs. C12 was washed out and replaced by fresh media containing either 1 μm NE, 40 μm Atglistatin, or a combination of both. After 24 h media was collected to measure cellular glycerol release, and cells were collected for lipid extraction and analysis by thin‐layer chromatography (TLC). (G) Representative thin‐layer chromatography (TLC) plate of lipids extracted from primary brown treated as described in (F). C12 alone was loaded as control for free fatty acid. (H) Quantification of free C12/TAG from TLC samples shown in (G), as a measure or lipolysis. Data are expressed as mean ± S.E.M. and represent *n* = 4 individual experiments. *P < 0.05 by Two‐way ANOVA. (I) Quantification of glycerol released into the media from *n* = 4 individual experiments. Data are expressed as mean ± S.E.M. **P < 0.01, *P < 0.05 by Two‐way ANOVA. Illustrations were created in BioRender.

We next developed a live‐cell assay using fluorescently‐labeled fatty acids to measure their mobilization from lipid droplets and assess the impact of MASh deficiency on adrenergically stimulated lipolysis, a key function of brown adipocytes. Brown adipocytes were pulsed with BODIPY‐C12 for 16 h, washed, and chased over 9 h while neutral lipids were labeled with BODIPY 493/503. Imaging was performed using high‐content confocal fluorescence microscopy (Fig. 5A). Control experiments validated the assay by showing that the BODIPY‐C12 signal loss was accelerated by the lipolysis activators NE and forskolin, completely blocked by the ATGL inhibitor Atglistatin, and unaffected by the fatty acid oxidation inhibitor etomoxir, confirming the specific measurement of lipolysis (Fig. 5B,C). Applying this assay to *Aralar 1* KD cells completely abrogated NE‐stimulated lipolysis compared to controls, consistent with a defect in acute lipolytic activation (Fig. 5D,E). To further validate this observation, we performed a longer pulse‐chase assay with BODIPY‐C12, followed by lipid extraction and TLC (Fig. 5F). Under NE treatment, control cells showed increased free fatty acid (FFA)‐to‐TAG ratios, indicative of lipolysis, whereas this response was lower in *Aralar 1* KD cells (Fig. 5G,H). These measurements truly reflect lipolysis as the presence of Atglistatin strongly reduces FFA‐to‐TAG ratios in both conditions (Fig. 5H). Finally, glycerol release into the media was used as a secondary measure of lipolysis. NE treatment strongly increased glycerol release in control cells, but this response was partially blunted in *Aralar 1* KD adipocytes (Fig. 5I). Interestingly, glycerol release remained lower in *Aralar 1* KD cells than in controls even in the presence of Atglistatin, suggesting that MASh inhibition engaged alternative pathways of glycerol utilization (Fig. 5I).

## Discussion

The malate–aspartate shuttle (MASh) is well characterized in metabolically active tissues such as the liver and heart, but its physiological role in thermogenic BAT remains largely unexplored [14, 15, 16, 17, 18, 19, 20, 21]. Here we provide direct evidence that MASh is functional in brown adipocytes and supports lipolysis, especially under adrenergic stimulation. Our findings reveal a previously unrecognized role for the MASh in regulating lipid metabolism in brown adipocytes, extending its classical function in maintaining cytosolic NAD^+^ redox balance in other tissues.

While direct evidence for MASh function in BAT is emerging, several studies have underscored its physiological relevance [18, 19, 20, 21]. Early research in rat BAT demonstrated that extramitochondrial NADH oxidation provided by MASh activity increased in response to a low‐protein diet [18]. Similarly, cold exposure has been reported to upregulate the expression of key enzymes involved in cytosolic NAD^+^ regeneration, including MDH1/2 and GOT1 (MASh components), cG3PDH and mG3PDH (GPSh components), and LDH [18]. Together, these findings suggest that BAT recruits multiple cytosolic NAD+ –regenerating pathways to sustain the high glycolytic flux required during thermogenic activation, when the GAPDH reaction continuously demands NAD^+^. Our results, supported by recent literature, strongly indicate that the MASh plays a key role in facilitating fatty acid‐dependent thermogenesis in brown adipocytes. Specifically, BAT‐targeted overexpression of GOT1 has been shown to enhance β‐oxidation and support acute cold‐induced thermogenesis [20]. Interestingly, genetic ablation of GOT1—and thus MASh inhibition—preserves cold‐induced thermogenesis by promoting a metabolic shift from fatty acid to glucose oxidation [20]. Our findings corroborate and extend these observations by demonstrating that MASh impairment sustains overall respiratory activity in NE‐stimulated brown adipocytes (Fig. 3A–D), while concurrently impairing lipolysis and resulting in an accumulation of small lipid droplets (Figs 4 and 5). Collectively, these data suggest that MASh not only modulates substrate preference toward fatty acid oxidation but also facilitates lipolysis, an essential upstream step that enables lipid oxidation and supports thermogenic heat production.

Mechanistically, the connection between the MASh and lipolysis appears to involve regulation of the cytosolic NAD^+^/NADH redox balance. MASh activity facilitates the regeneration of NAD^+^ from NADH in the cytosol, primarily through the reduction of oxaloacetate to malate by cytosolic malate dehydrogenase (Fig. 1G,H). Despite the theoretical expectation that reductions in MASh activity would disturb redox homeostasis, our metabolomic data show that the lactate/pyruvate ratio remains unchanged under conditions of MASh impairment, indicating that the overall cytosolic NAD^+^/NADH ratio is maintained (Fig. 2G–I). While direct measurements of cytosolic NAD^+^/NADH were not performed, the preserved lactate/pyruvate ratio in *Aralar 1* KD cells under basal conditions strongly suggests redox stability, likely due to compensatory activity by alternative mitochondrial shuttles or metabolic adaptations that maintain NAD redox homeostasis despite MASh impairment [20, 31].

Previous studies have shown that BAT exhibits high activity of mitochondrial FAD‐dependent glycerol‐3‐phosphate dehydrogenase (mG3PDH), which functions as an electron sink to sustain low cytosolic NADH levels essential for continuous glycolytic flux [11]. Accordingly, suppression of the MASh, either genetically or pharmacologically, is likely to induce a compensatory upregulation of the GPSh. This adaptation would enhance G3P turnover, contributing to the maintenance of cytosolic NAD redox balance. Moreover, the increased flux through the GPSh could favor fatty acid esterification and triglyceride synthesis or re‐esterification, consistent with our findings in *Ogc* and/or *Aralar 1* KD cells, where (i) triglyceride content rises (Fig. 4K,M), (ii) overall respiratory rates remain largely unaltered (Fig. 3A–D), and (iii) glycerol release declines significantly (Fig. 5I). Notably, the decrease in glycerol release persists even when lipolysis is blocked by Atglistatin, suggesting that the available G3P pool is rerouted from dephosphorylation and extracellular release toward oxidation to DHAP by mG3PDH to regenerate cytosolic NAD^+^ under MASh‐deficient conditions. We propose that interference with the MASh does not directly impact lipolysis but instead alters the cellular balance between DHAP and G3P owing to enhanced activity of the GPSh. This metabolic shift would favor the esterification of G3P with free fatty acids, thereby promoting triglyceride synthesis. These results support the notion that, even during adrenergic stimulation—when long‐chain unsaturated fatty acids and their CoA esters strongly inhibit mG3PDH activity [11]—the residual flux through the glycerophosphate shuttle remains critical for sustaining cytosolic NAD redox equilibrium [11, 21, 32].

Our results also demonstrate an increased number of small lipid droplets during MASh disruption (Fig. 4B,F), which may impair ATGL‐mediated hydrolysis of intra‐cellular triglycerides. This is explained by the fact that ATGL has been shown to preferentially act on larger lipid droplets [33]. Moreover, the accumulation of triglycerides may reflect passive re‐esterification of fatty acids that were not mobilized due to impaired hydrolysis, rather than an active increase in *de novo* esterification. Notably, *Atgl* mRNA measurement in our study was primarily used as a marker of brown adipocyte differentiation, rather than as a direct indicator of ATGL protein abundance or enzymatic activity. We detected increased *Atgl* expression only in *Ogc* KD cells (Fig. 2K), but not in *Aralar 1* KD cells (Fig. 2J). This likely does not reflect a major difference in differentiation status, as other brown adipocyte markers and NE‐stimulated respiration were comparable between scramble and knockdown cells (Figs 2J,K and 3A–D). Although lipolysis was not evaluated in *Ogc* KD cells, basal lipolysis remained unchanged in *Aralar 1* KD cells while NE‐stimulated lipolysis was delayed or partially inhibited (Fig. 5D,E,G–I). Notably, the enhanced fatty acid esterification observed in *Ogc* KD cells despite elevated Atgl expression is not contradictory, since in brown adipocytes lipolysis and re‐esterification occur concurrently to sustain high lipid turnover [34].

Phenotypes observed in *Aralar 1* KD cells closely resemble those in *Ogc* KD cells, particularly in terms of lipid metabolism alterations and energy expenditure. The main difference lies in mitochondrial morphology, which is altered in *Ogc* KD cells but remains unchanged in *Aralar 1*‐silenced cells (Fig. 3G,J). Unlike *Ogc*, which lacks an alternative isoform, *Aralar 1* has *Citrin* (or SLC25A13) as a paralog that may partially compensate for its loss. This potential compensation might explain the preservation of mitochondrial morphology in *Aralar 1* KD cells. Nonetheless, our metabolomics data demonstrate that downregulation of *Aralar 1* alone significantly reduces the aspartate/glutamate ratio (Fig. 2D–F). Since this ratio reflects glutamate catabolism, its decrease indicates impaired malate–aspartate shuttle activity and reduced glutamate catabolism. Therefore, although compensation by *Citrin* cannot be entirely excluded, *Aralar 1* KD alone suffices to cause substantial impairment of malate–aspartate shuttle function. It is also important to note that mitochondrial morphology was quantified using MTDR in *Aralar 1* KD cells and TMRE in *Ogc* KD cells due to experimental constraints (see Methods). TMRE is a membrane potential–dependent dye, which could potentially influence morphology measurements. To minimize this risk, we used confocal microscopy, which reduces the likelihood of pixel expansion due to higher fluorescence intensity, and set thresholds to detect even weakly stained mitochondria. Nonetheless, we cannot fully exclude the possibility that the differences in morphology observed between *Aralar 1* and *Ogc* KD are influenced by using different dyes; however, statistical comparisons were never performed across samples stained with different dyes.

Our previous work established that inhibiting MPC in brown adipocytes triggers a compensatory activation of the MASh, which in turn increases energy expenditure [21]. Intriguingly, MPC inhibition also stimulates lipid cycling and fatty acid utilization. Complementing these findings, genetic deletion of the MASh component GOT1 has been shown to produce the opposite effect: an upregulation of glucose uptake, glycolysis, and expression of the mitochondrial pyruvate carriers MPC1 and MPC2 [20]. Supporting these observations, an early study demonstrated that pharmacological inhibition of MASh increases the lactate/pyruvate ratio as a compensatory attempt to maintain cytosolic NAD^+^ which then limits the mitochondrial pyruvate metabolism [28]. This creates a compelling picture of a reciprocal feedback mechanism. Blocking the mitochondrial pyruvate metabolism activates MASh, while blocking the MASh enhances mitochondrial pyruvate metabolism. Therefore, MASh appears to function as a metabolic switch, mediating a preference for fatty acid oxidation when active and for glucose metabolism when its function is limited, as recently demonstrated [20].

The findings presented here suggest that disrupting the MASh would activate the GPSh, a pathway normally suppressed in BAT during NE stimulation [11]. Interestingly, cG3PDH overexpression activates GPSh, increasing lipid content in BAT by converting DHAP to G3P and thus supplying substrates for triglyceride synthesis even under redox imbalance [35]. Increased availability of G3P, coupled with reduced FFA oxidation, could shift the metabolic balance toward lipid storage. The hypothesis of the GPSh acting as a compensatory pathway for NAD^+^ regeneration is supported by studies in GOT1‐deficient BAT, where it is required to maintain glycolysis during thermogenic challenges [20]. This same compensatory mechanism would explain the reduced glycerol release we observed in *Aralar 1* KD brown adipocytes (Fig. 5I). We propose that by blocking the MASh, *Aralar 1* silencing engages the GPSh, which increases the cellular demand for its substrate, G3P. The cell meets this demand by phosphorylating the free glycerol pool, thereby limiting its release. It is important to note that while glycerol release is commonly used as a proxy for lipolysis, it may underestimate total lipid turnover. This measurement primarily captures the complete hydrolysis of triglycerides into FFAs and glycerol, without accounting for partial hydrolysis or re‐esterification processes. Importantly, glycerol can originate not only from TAG breakdown in LDs, but also from the hydrolysis of intermediates such as monoacylglycerols (MAGs), or from incomplete esterification during *de novo* lipogenesis [36]. Supporting this, we observed that Atglistatin, an inhibitor of ATGL that blocks the initial step of lipolysis, was unable to fully suppress NE‐stimulated glycerol release (Fig. 5I), despite effectively preventing TAG degradation (Fig. 5D–H). This discrepancy suggests that a fraction of glycerol release may arise from ongoing lipid cycling rather than direct lipolysis. Recent work from our group and others has highlighted that brown adipocytes engage in active lipid cycling, wherein fatty acids are continuously hydrolyzed and re‐esterified [21, 37]. This cycle is metabolically demanding and depends on sustained cytosolic NAD^+^ availability to drive glycolysis and G3P synthesis. The reduced glycerol release observed in MASh‐deficient cells might thus reflect a broader disturbance in lipid turnover rather than just lipolysis itself. Another potential mechanism that might be engaged as a compensatory alternative for providing cytosolic NAD^+^ is the enzyme Aimf2, identified as a novel and BAT‐specific external NADH dehydrogenase‐like enzyme that mediates cytosolic NADH oxidation, glycolysis and thermogenesis in BAT [38]. However, its involvement in the context of MASh inhibition needs to be elucidated.

## Conclusion

In brown adipocytes, functional MASh is essential for efficient thermogenic lipolysis. Disrupting the MASh triggers a compensatory increase in mitochondrial content, but these new organelles are metabolically reprogrammed, resulting in lower oxygen consumption per mitochondrion. Importantly, thermogenically activated lipolysis in brown adipocytes depends on a functional MASh. Therefore, our data position MASh as a central player in coordinating cytosolic redox state with lipid mobilization in BAT. By linking NAD^+^ recycling to lipolytic capacity, MASh supports the metabolic flexibility required for efficient thermogenesis, which depends on the coordinated regulation of glucose and fatty acid metabolism in brown adipocytes. Although the precise mechanisms connecting MASh activity and lipolysis remain to be elucidated, our data show that disrupting MASh impairs lipolysis, highlighting its critical role in BAT metabolism. These findings highlight a redox–lipid axis that could be targeted to enhance BAT function in metabolic disorders such as obesity and insulin resistance.

## Materials and methods

### *In silico* protein level analysis

Protein levels of the MASh components were analyzed in murine intact brown adipocytes, liver [13], and BAT isolated mitochondria [12]. Expression values represent the ratio Heavy/Light in quantitative proteomics using SILAC mice.

### Animals

The investigation with experimental animals was approved by the Experimental Animal Ethics Committee of the Federal University of Rio de Janeiro under the protocol A09/17–050‐16 and by the ARC/IACUC under the protocol ARC‐2017‐019 of the University of California, Los Angeles. 4‐ to 5‐week‐old wild‐type male C57Bl/6J mice (from either Cemib‐UNICAMP, Brazil or Jackson Laboratory, Bar Harbor, ME, USA) were fed a standard chow diet and kept under controlled conditions (19–22 °C and a 14: 10 h light–dark cycle) until euthanasia performed by asphyxiation in a CO_2_ atmosphere and subsequent cardiac puncture. All experiments were performed in accordance with the Guide for Care and Use of Laboratory Animals of the NIH (NIH Publications No. 8023, revised 1978).

### Mitochondrial isolation

Interscapular and subscapular brown adipose tissues were dissected and deposited in a small volume of ice‐cold SHE buffer (sucrose 250 mm; HEPES 5 mm; EGTA 2 mm, pH 7.2). Pieces were then gently homogenized in a 15 mL Potter‐Elvehjem tissue grinder in a Teflon pestle with 10 mL of either ice‐cold SHE buffer for downstream enzymatic measurements or in SHE buffer +2% fatty‐acid‐free bovine serum albumin for shuttle activity assessment. The homogenate was filtered through two layers of gauze and centrifuged at 8500 × ***g*** for 10 min at 4 °C. Following centrifugation, the cytosol‐containing supernatant was removed and reserved, and the mitochondrial‐containing pellet was resuspended in SHE buffer. Mitochondrial isolation was performed as previously described [39], and protein concentration was determined using a BCA protein assay (Thermo Fisher Scientific, Roskilde, Denmark) or the Folin method [40].

### Mitochondrial enzyme activities

Glutamic oxaloacetic transaminase (GOT) activity was measured in cytosolic (GOT1) and mitochondrial (GOT2) fractions as described [41]. Malate dehydrogenase (MDH) activity was measured in cytosolic (MDH1) and mitochondrial (MDH2) fractions by adding the sample (100 μg of protein) to a quartz cuvette containing 89 mM Tris pH 7.8, 5 μm rotenone, and 160 μm NADH. Reaction was started by adding 5 mm oxaloacetate, and NADH absorbance (340 nm) was measured by a Shimadzu UV‐72550 spectrophotometer (Shimadzu Scientific Instruments, Japan).

### Assessment of MASh activity

Mitochondria were incubated with or without 2.5 mm fluorocitrate for 10 min at 4 °C. 200 μg of mitochondrial protein was added to a quartz cuvette containing 1 mL of MSK buffer (75 mm mannitol; 25 mm sucrose; 5 mm K_2_PO_4_; 20 mm Tris/HCl; 0.5 mm EDTA; 100 mm KCl; 0.1% BSA; pH 7.4), 4 U·mL^−1^ GOT, 6 U·mL^−1^ MDH, 5 mm aspartate, 5 mm malate, 0.5 mm ADP, 200 nm ruthenium red, 8 μm CaCl_2_ and 0.25 mM fluorocitrate at 37 °C and MASh activity was assessed by monitoring NADH fluorescence and calculating NADH oxidation rates, as previously described [23, 24].

### Primary brown adipocyte culture

Primary brown adipocytes were isolated and cultured as described [21, 42]. In brief, BAT tissue was dissected from interscapular, subscapular, and cervical regions of four male mice and digested in 1.5 mg·mL^−1^ collagenase Type II (Worthington, Lakewood, NJ). The tissue was then filtered through a 100 μm and 40 μm mesh and centrifuged. Cells were resuspended in brown adipocyte culture media (25 mm glucose DMEM supplemented with 10% newborn calf serum (Sigma‐Aldrich, St. Louis, MO), 4 mm glutamine, 10 mm HEPES, 0.1 mg·mL^−1^ sodium ascorbate, 100 U·mL^−1^ penicillin, 100 μ·mL^−1^ streptomycin) and plated in a 6‐well plate. Pre‐adipocytes were incubated in a 37 °C, 8% CO_2_ for 72 h and then lifted using STEMPro Accutase (Thermo Fisher Scientific, Roskilde, Denmark), counted, and re‐plated in the final experimental vessel. 24 h later differentiation was induced by switching the media to BAT differentiation media (growth media supplemented with 1 μm rosiglitazone maleate (Sigma‐Aldrich, St. Louis, MO) and 4 nm human insulin (Humulin R, Eli Lilly, Indianapolis, IN)). Cells were differentiated for 7 days and media changed every other day. The primary pre‐adipocytes used in this study were freshly isolated and only maintained in culture for about 10 days during differentiation into brown adipocytes. Cultures were handled under standard sterile conditions, and all cells behaved as expected in terms of morphology and functional markers. Given the short culture period, routine mycoplasma testing was not performed, as the risk of contamination affecting the results was considered minimal.

### Gene silencing

Gene silencing was achieved using two complementary approaches: adenoviral‐mediated transfection and siRNA‐mediated transfection. Experimental procedures were based on protocols previously developed by our group, with minor adaptations in the adenoviral transduction step [21].

For adenoviral‐mediated gene silencing, on day 3 of differentiation, adipocytes were incubated with 1.5 μL·mL^−1^ of adenoviral preparation (10^9^ particles per mL) for 24 h in BAT differentiation medium containing 1 μg·mL^−1^ polybrene (hexadimethrine bromide, Sigma‐Aldrich, St. Louis, MO). Adenovirus‐containing medium was removed 24 h later, and cells were maintained in differentiation medium until day 7 of differentiation when experiments were performed. The adenoviral constructs Ad‐mSLC25A11 (Ogc) and Ad‐mKate2 shControl were generated by and purchased from Welgen (Worcester, MA).

For siRNA‐mediated gene silencing, undifferentiated pre‐adipocytes were subjected to transfection with either scramble RNA or *Aralar 1* (*Slc25A12*) siRNA. Transfections were performed using Lipofectamine RNAiMAX (Thermo Fisher Scientific, Roskilde, Denmark), according to the manufacturer's protocol. Cells were incubated with Opti‐MEM medium (Thermo Fisher Scientific, Roskilde, Denmark) and Lipofectamine reagent with a final siRNA or scramble RNA concentration of 100 nm. After a 4 h incubation, DMEM with 1% fetal bovine serum (Thermo Fisher Scientific, Roskilde, Denmark) was added, and the cells were incubated overnight. On the following day, the transfection medium was replaced with a differentiation medium, and experiments were conducted on day 7 of differentiation. The siRNA reagents used included ON‐TARGETplus Non‐targeting Pool (D‐001810‐10‐05) and ON‐TARGETplus Mouse *Slc25a12* siRNA (L‐064268‐01‐0005) from Dharmacon (Lafayette, CO).

### Respirometry

Respirometry analyses were performed based on protocols previously established by our group [21], with minor adaptations. Seahorse experiments were performed on day 7 of differentiation. Prior to respirometry measurements, culture media was replaced with respirometry media Seahorse XF Base medium (Agilent Technologies, Santa Clara, CA) supplemented with 5 mm glucose and 3 mm glutamine when indicated, and cells were incubated for 30–45 min at 37 °C (without CO_2_). When indicated, additional supplements were included as specified in the experimental design. The ports of the Seahorse cartridge were loaded with the compounds to be injected during the assay (50 μL per port) and the cartridge was calibrated. Oxygen consumption rates were measured using the Seahorse XF24‐3 extracellular flux analyzer (Agilent Technologies, Santa Clara, CA). The following compounds were used for injections during the assay: 1 μm norepinephrine (Levophed), 4 μm oligomycin A (Calbiochem, San Diego, CA), 40 μm etomoxir (Sigma‐Aldrich, St. Louis, MO), 4 μm antimycin A (Sigma‐Aldrich, St. Louis, MO). After the assay, cells were fixed using 4% paraformaldehyde (Thermo Fisher Scientific, Roskilde, Denmark). To normalize the data for possible differences in cell number, nuclei were stained with 1 μg·mL^−1^ Hoechst 33342 (Thermo Fisher Scientific, Roskilde, Denmark) and nuclei were counted using the Operetta High‐Content Imaging System (PerkinElmer, Waltham, MA).

### Live‐cell imaging

#### Super‐resolution confocal microscopy

Super‐resolution live‐cell imaging was performed on a Zeiss LSM880 using a 63× Plan‐Apochromat oil‐immersion lens and AiryScan super‐resolution detector with a humidified 5% CO_2_ chamber on a temperature‐controlled stage (37 °C). Cells were differentiated in glass‐bottom confocal plates (Greiner Bio‐One, Kremsmünster, Austria). Prior to imaging, cells were stained with 1 μm BODIPY 493/503 (BODIPY) (Thermo Fisher Scientific, Roskilde, Denmark) and 500 nm MitoTracker deep red (MTDR) (Thermo Fisher Scientific, Roskilde, Denmark) for 1 h, or 15 nm TMRE for 30 min. MTDR was washed out and cells were imaged in regular culture media in the presence of BODIPY. Cells stained with TMRE were measured in the presence of TMRE. BODIPY was excited with a 488 nm laser, MTDR was excited with a 633 nm laser, and TMRE was excited with a 552 nm laser. TMRE was used for *Ogc* KD cells because the scramble control for the shRNA viruses carries an mKate fluorescent tag, which overlaps with MTDR fluorescence, preventing its use. MTDR was used for *Aralar 1* KD cells. Image analysis was performed in FIJI (ImageJ, NIH). For the quantification of mitochondrial morphology and area, images stained with TMRE or MTDR were analyzed. Thresholds were adjusted to ensure that even weakly stained mitochondria were detected and included in the analysis. Only the mitochondrial area was evaluated, independent of fluorescence intensity. Image contrast and brightness were not altered in any quantitative image analysis protocols. For representative images, brightness and contrast were equivalently modified in the different groups compared, to allow proper representative visualization of the effects revealed by unbiased quantitation. Detailed image analysis protocols are available upon request.

#### High‐content imaging of lipolysis

Lipolysis imaging was performed on an Operetta High‐Content Imaging System (PerkinElmer, Waltham, MA) using a 20× lens with a humidity‐controlled cell culture incubator (5% CO_2_ and 37 °C). Cells were differentiated in a black‐walled, clear‐bottom 96‐well microplate. On the 6th day of differentiation, cells were incubated with 1 μm BODIPY 558/568 C12 (BODIPY C12) (Thermo Fisher Scientific, Roskilde, Denmark) overnight. The next day, BODIPY C12 was washed out and cells were stained with BODIPY 488/503. Cells were incubated with the following compounds: 10 μm Forskolin (Sigma‐Aldrich, St. Louis, MO); 10 μm etomoxir (Sigma‐Aldrich, St. Louis, MO); 40 μm Atglistatin (Selleck Chemicals, Houston, TX); 1 μm norepinephrine (Levophed). Cells were imaged immediately after addition of compounds for 5–9 h, taking one image per hour. Analysis was performed using the Operetta integrated software. Lipid droplet area was defined based on BODIPY 493/503 signal, which was used to generate a mask identifying all lipid droplets. Within this mask, the mean fluorescence intensity of BODIPY C12 was quantified over time to monitor the release of previously esterified C12. To account for variability between samples, data were normalized to each sample's individual baseline at time point 0 and expressed as fold change relative to this baseline.

### Thin‐layer chromatography of intra‐cellular lipids

Cells were seeded and differentiated in 12‐well plates. For measurements of fatty acid esterification, cells were incubated with 1 μM BODIPY 558/568 C12 and 1 μm NE (when indicated) for 24 h. For measurements of lipolysis, cells were incubated with 1 μm BODIPY 558/568 C12 overnight. The next day, BODIPY C12 was washed out, and cells were treated with 40 μm Atglistatin or 1 μm NE (as indicated) for 6 h. Thin‐layer chromatography of intra‐cellular lipids [43] with minor modifications. Intra‐cellular lipids and lipids from media were extracted in 500 μL chloroform. Chloroform was evaporated using the Genevac EZ‐2 Plus Evaporating System (Genevac, Ipswich, United Kingdom). Lipids were then dissolved in 10 μL chloroform, and 1 μL was spotted on a TLC plate (silica gel on TLC Al foils, Sigma‐Aldrich, St. Louis, MO). Lipids were resolved based on polarity in a developer solution containing ethyl acetate and cyclohexane in a 2 : 1 ratio. TLC plates were imaged on the ChemiDoc MP imaging system (Bio‐Rad Laboratories, Hercules, CA). Band densitometry was quantified using FIJI (ImageJ, NIH).

### Total glycerol assessment in media

Experiments were performed with media from TLC experiments assessing lipolysis. Total glycerol released into the media was measured using Free Glycerol Reagent (#F6428; Sigma‐Aldrich, St. Louis, MO) according to the manufacturer's instructions.

### Gene expression

Primary brown adipocytes transduced with the adenovirus (*Ogc*‐KD) or transfected siRNA (*Aralar 1*‐KD) were collected on the 7th day of differentiation. RNA extraction was performed using the Direct‐zol RNA Miniprep Plus Kit^®^ (Zymo Research, Irvine, CA) according to the manufacturer's instructions. 1 μg of RNA was used for cDNA synthesis by the High‐Capacity cDNA Reverse Transcription Kit^®^ (Applied Biosystems, Foster City, CA). Then, qPCR was performed with 0.4 ng·μL^−1^ cDNA and 240 nm of each primer. Gene expressions were normalized by an endogenous control gene (36b4). Primers are listed in Table 1.

**Table 1:** Primers used for qPCR.

Gene	Forward primer 5′‐3′	Reverse primer 5′‐3′
*Aralar 1*	AGT CTC CTC TTG GGT GTT AGA	CTT CTG CTT TCT CCT GTC TCC
*Ogc*	TGGTTACCTACGAGCTTCTGC	ACCGATGTGATCGGGGTTG
*UCP1*	GGC CTC TAC GAC TCA GTC CA	TAA GCC GGC TGA GAT CTT GT
*Atgl*	TCC GAG AGA TGT GCA AAC AG	CTC CAG CGG CAG AGT ATA GG
*Pgc1α*	AAG ATC AAG GTC CCC AGG CAG TAG	TGT CCG CGT TGT GTC AGG TC
*Tfam*	CAC CCA GAT GCA AAA CTT TCA	CTG CTC TTT ATA CTT GCT CAC AG
*Elovl3*	TCC GCG TTC TCA TGT AGG TCT	GGA CCT GAT GCA ACC CTA TGA
*Prdm16*	GCC ATG TGT CAG ATC AAC GA	CCT TCT TTC ACA TGC ACC AA
*36b4*	GTC ACT GTG CCA GCT CAG AA	TCA ATG GTG CCT CTG GAG AT

### Metabolomics

For metabolomics experiments, cells were cultured and differentiated in 6‐well plates using BAT differentiation media. siRNA transfection was done on day 3 of differentiation. On day 7 of differentiation, cells were collected for metabolite extraction. Metabolite extraction and GC/MS were performed as previously described in detail [44].

### Statistical analyses

Data were presented as mean ± SEM for all conditions. Comparisons between groups were done by one‐way ANOVA with Tukey's or Holm Sidak's test for pairwise multiple comparisons. When appropriate, two‐way ANOVA with Tukey's multiple comparisons test was employed. Pairwise comparisons were carried out by two‐tailed Student's *t*‐test. Differences of *P* < 0.05 were considered to be significant. All graphs and statistical analyses were performed using graphpad prism 10 for Windows (graphpad software, San Diego, CA).

## Author contributions

Conceptualization; CMF, MV, ML, MFO, Investigation; MV, CMF, FV, AB, GSF, KM, AEJ, ASD, RAP, LS, MFO, Writing‐Original Draft; MV, CMF, MFO. Writing‐Review & Editing; MV, CMF, AB, RAP, AEJ, ASD, MFO, OSS, ML.

## Conflict of interest

The authors declare no conflict of interest.

## Data Availability

The complete set of raw data will be made available, upon reasonable request, by the corresponding author.
